# Study on enzyme activities and metabolomics of two *Penicillium chrysogenum* strains during fermentation of soybean paste

**DOI:** 10.3389/fmicb.2025.1570939

**Published:** 2025-05-27

**Authors:** Xiaodong Sun, Hong Yang, Guangwei Huo, Linlin Li, Guozhong Lyu

**Affiliations:** College of Environment and Resources, Dalian Minzu University, Dalian, China

**Keywords:** *Penicillium chrysogenum*, single strain fermentation, protease activity, lipase activity, metabolomics

## Abstract

In this study, two strains of *Penicillium chrysogenum* JSPP6_1 and GJY1_3 were isolated from naturally fermented soybean paste. In order to understand the potential role of the two strains in the fermentation of soybean paste, both strains were used for fermenting soybean paste, respectively. The fermentation period was 45 days, with samples taken every 5 days. Protease activities of GJY1_3 and JSPP6_1 reached their maximum value on the 15th day of fermentation, 108.95 ± 6.38 and 70.79 ± 2.13 U/mL, respectively. Lipase activities of JSPP6_1 reached the maximum at the 15th day of fermentation (14.52 ± 0.68 U/mL), while that of GJY1_3 reached the maximum at the 20th day of fermentation (7.41 ± 0.75 U/mL). Samples were taken for the metabolomic study on day 15 of fermentation. Fifty-three different metabolites were obtained, of which 33 were known, including seven amino acids, 18 organic acids and anhydrides, as well as a small amounts of sugars, glycolic acids and alcohols. Seventeen metabolic pathways were identified, of which six pathways were significant. The results showed that JSPP6_1 could produce more lipase, and the relative levels of various organic acids were higher in JSPP6_1. GJY1_3 may produce more protease, the relative contents of various amino acids being very higher in GJY1_3, including glutamic acid and glutamine, which are flavor amino acids. Both strains showed obvious functional differences in fermenting soybean paste.

## 1 Introduction

*Penicillium chrysogenum*, a ubiquitous species in the *Penicillium* genus, is widely distributed in air, soil, and diverse substrates. Its primary industrial application remains penicillin production (Barreiro and García-Estrada, 2018; Barreiro et al., 2012). The strain’s industrial significance originated from its 1943 isolation from a moldy watermelon in Peoria, Illinois (USA), which exhibited higher penicillin titers than predecessors and became the progenitor of modern industrial strains (Bennett and Chung, 2001; Gaynes, 2017). Over decades, advancements in strain optimization, including fermentation condition refinement, mutagenesis, and high-yield strain screening—have significantly enhanced penicillin productivity (Cantoral et al., 1987; Beri and Turner, 1987; Sánchez et al., 1987; Barredo et al., 1989a, 1989b; Li, 2015). Recent studies further revealed diverse secondary metabolites in *P. chrysogenum*, such as terpenoids and phenolic acids from Antarctic marine isolates, and ergosterol with antifungal activity via solid-state fermentation (Qiao et al., 2020; Qiao et al., 2017; Li et al., 2022; Zhang, 2009; Jakovljević et al., 2014). *Penicillium chrysogenum* has become a pivotal fungal species in food biotechnology, attributed to its ability to produce a wide range of enzymes (e.g., lipase, amylase, protease, β-mannanase, hemicellulase) and organic acids. These enzymatic systems facilitate its utilization in food fermentation processes, such as tea processing, where it modulates biochemical compositions and reshapes microbial community structures. Moreover, *P. chrysogenum* is widely applied in meat fermentation (e.g., duck products) to develop unique flavor and texture characteristics while improving product quality, microstructural integrity, and physicochemical stability (Bancerz et al., 2005; Ferrer et al., 2000; Ertan et al., 2014; Guo et al., 2021; Zhen et al., 2021; Liu et al., 2021; Long et al., 2020; Lan et al., 2020; Donato et al., 2017).

Soybeans are a nutrient-dense food, containing approximately 36–40% protein, 18–20% fat, 30% carbohydrates, and 9–13% dietary fiber, as well as essential micronutrients including calcium, iron, and B vitamins (Chen, 2008). During the fermentation for producing soybean paste, the protein is hydrolyzed into smaller amino acids that enhance bioavailability, while the fat is transformed into fatty acids and aromatic compounds, which are more readily absorbed and utilized by the human body. Two strains of *Penicillium chrysogenum* were isolated from fermented soybean paste under natural conditions in rural areas of Northeast China. In order to understand the role of *P. chrysogenum* in soybean paste fermentation and the metabolites produced, single strain fermentation was used to ferment soybean paste. The fermentative capacity of two strains was evaluated by analyzing protease and lipase activities, as well as conducting metabolomics studies. The findings provided a theoretical basis for screening of functional strains of the soybean paste fermentation, a better understanding of the functions of *P. chrysogenum* and an enrichment of the reserves of the strains.

## 2 Materials and methods

### 2.1 Strain activation

Took the two strains of *Penicillium chrysogenum* from the refrigerator, inoculated them onto PDA plates for activation, and incubated them at 28°C in the dark for 7 days until sporulation occurred. Transferred a small amount of the colonies to an appropriate volume of sterile water to prepare a spore suspension with a concentration of 10^6^ CFU/mL for subsequent fermentation inoculation.

### 2.2 Soybean paste fermented by single strain

Soaked the soybeans overnight, then weighed out 120 g of soaked soybeans and transferred them into a triangular flask. Sterilized at 121°C for 60 min. Inoculated the soybeans with 1 mL of spore suspension from each fungus and incubated in the dark at 28°C for 1 week until sporulation occurred. Once the spores fully covered the surface of the soybeans, added 60 mL of 10% (w/v) saline solution to the flask and fermented the mixture for 45 days at room temperature. Repeated this procedure six times for each species. Collected samples every 5 days to measure a series of indicators (Sun et al., 2019).

### 2.3 Determination of protease and lipase activity

Five grams of sample was taken from fermented soybean paste every 5 days, and was added 50 mL of sterile deionized water, dispersed evenly, shaken and cultured at 30°C for 4 h, and then stood still. The filtrate was centrifuged at 4,200 rpm for 20 min, and the supernatant was used as a crude enzyme to determine protease and lipase activity.

#### 2.3.1 Determination of protease activity

The standard curve of tyrosine was designed and protein activity was determined by the foline-phenol method (Chen, 2008; National Standardization Administration Committee, 2023). First, different concentrations of tyrosine standard solution were prepared. Took 1 mL each, added 5 mL of 0.4 mol/L sodium carbonate and 1 mL of foline-phenol working solution respectively, shook them well and placed them in a water bath, kept them at 40°C for 20 min, and measured the absorbance value at the wavelength of 680 nm with a spectrophotometer. The standard curve was draw with the absorbance value (*y*) as ordinate and the mass concentration of tyrosine (*x*) as abscissa.

One millilitre of crude enzyme solution was taken, reacted at 40°C for 2 min, added casein and shaken well, continued to keep at 40°C for 10 min, added 2 mL of TCA (trichloroacetic acid) and shaken well. Then, it was taken out and left on for 10 min for filtration. One millilitre of filtrate was taken, added 5 mL of sodium carbonate and 1 mL of foline-phenol reagent, shaken well, kept at 40°C for 20 min, and measured the absorbance at the wavelength of 680 nm. In the blanked control, TCA 2 mL was added first, followed by casein. The rest of the steps were the same. The measured absorbance value was substituted in the standard curve for tyrosine concentration. The calculation formula is as follows:


(1) $$U=\frac{C\times N\times V1\times 4}{10\times V}$$

*U* is the enzyme activity of the sample, U/mL (μmol/mL min); *C* is the mass concentration of tyrosine solution obtained from the standard curve, μg/mL; *N* is the dilution ratio of enzyme solution; 4 is the total volume of reaction reagent, mL; *V*_1_ is the volume of the crude enzyme solution used in the reaction, mL (1 mL); 10 is the reaction time, min; *V* is the total volume of enzyme solution, mL.

#### 2.3.2 Determination of lipase activity

The enzyme activity was measured with NaOH titration method (Yang and Zhang, 2017). Took a 100 mL conical flask and added 4 mL 0.025 mol/L phosphate buffer and 5 mL tributyl ester emulsion into it, put it in a 40°C water bath for 5 min, and then added 1 mL crude enzyme solution. Precise timing must begin with the addition of enzymatic solution, and the temperature must be maintained for 15 min. Then, it was taken out and immediately added 15 mL of 95% ethanol to stop the enzyme action. Three droplets of phenolphthalein indicator were added to the reaction solution and titrated in pink with 0.05 mol/L of NaOH standard solution. Prior to the addition of the enzymatic solution to the blank, 15 mL of 95% ethanol was added to inactivate the enzymatic solution. The formula for lipase activity is as follows:


(2) $$U=\frac{V-V0}{t\times n}\times M\times 1000$$

*U* is the lipase activity of the sample, U/mL (μmol/min mL); *V* is the volume of NaOH consumed by titration solution, mL; *V*_0_ is the volume of NaOH consumed for titrating the blank solution, mL; *t* is the reaction time, min; *n* is the volume of enzyme solution, mL; *M* is the concentration of NaOH solution for titration, mol/mL.

### 2.4 Metabolomics study

#### 2.4.1 Metabolite extraction and machine detection

Took 0.05 g soybean paste, put it into an EP tube, added liquid mixture of 0.4 mL of 75% methanol aqueous solution and 20 μL of adonitol (2 mg/mL stock in dH_2_O) as the internal standard, and shook for 30 s; then, the tube was homogenized in a ball mill at 45 Hz for 6 min, and centrifuged for 15 min at 4°C with 12,000 rpm; subsequently, 0.35 mL of the supernatant was transferred into a new GC/MS glass vial (Sun et al., 2019).

Dried the above solution in a vacuum concentrator, added 80 μL of pyridine solution of methoxyamine hydrochloride with the concentration of 20 mg/mL, and cultured at 80°C for 30 min; 100 μL of BSTFA regent (1% TMCS, v/v) was added to the liquid aliquots, incubated for 2 h at 70°C. In addition, the sample was mixed with 10 μL of FAMEs (a standard mixture of fatty acid methyl esters in chloroform), cooled to room temperature and shaken well for GC-MS analysis.

The samples were detected by a gas chromatograph of Agilent 7890, coupled with a Pegasus HT time-of-flight mass spectrometer. The chromatographic column used for detection is DB-5MS capillary column (J&W Scientific, Folsom, CA, United States), with inside diameter 30 m × 0.25 mm and film thickness 0.25 μm. Approximately 1 μL treated sample was injected in splitless mode. The carrier gas was helium with the front inlet purge flow 3 mL/min, and the gas flow rate through the column was 1 mL/min. Initially, the temperature was kept at 50°C for 1 min; it was then raised to 320°C at a speed of 10°C/min and held for 10 min. Injection, transmission line and ion source temperatures were 280°C, 280°C, and 250°C, respectively. In electron collision mode, the power output is −70 eV. The full-scan mode is the primary way to obtain the mass spectrometry data, with the m/z range from 85 to 600 and running rate of 20 spectra per second after 366 s solvent delay.

#### 2.4.2 Data analysis

Metabolites were identified through the retention time index match (RI) and mass spectrum match. Chroma TOF4.3X software (LECO Corporation) and LECO-Fiehn Rtx5 database were used to process data, including exacting of the raw peaks, peak alignment, peak identification, integration of the peak area, calibration of the baseline and the baselines filtering (Kind et al., 2009). Half of the minimum value was used to fill in the missing values from the raw data. In addition, the internal standard normalization method was also used in the data analysis. The first principal component of variable importance in the projection (VIP) was used to refine the analysis, and the metabolites were initially selected with the VIP values higher than 1.0. Subsequently, the student t-test (*p* < 0.05) was also used to evaluate variables. The quantitative value of the differential metabolites was calculated by the Euclidean distance matrix, and the differential metabolites were clustered by a complete linkage method and showed by a heatmap (Kolde, 2019). In addition, the differential metabolic pathways were found by the databases KEGG^1^ and NIST.^2^

## 3 Results and discussion

### 3.1 Difference in protease and lipase activities between two strains

**Figure 1:**
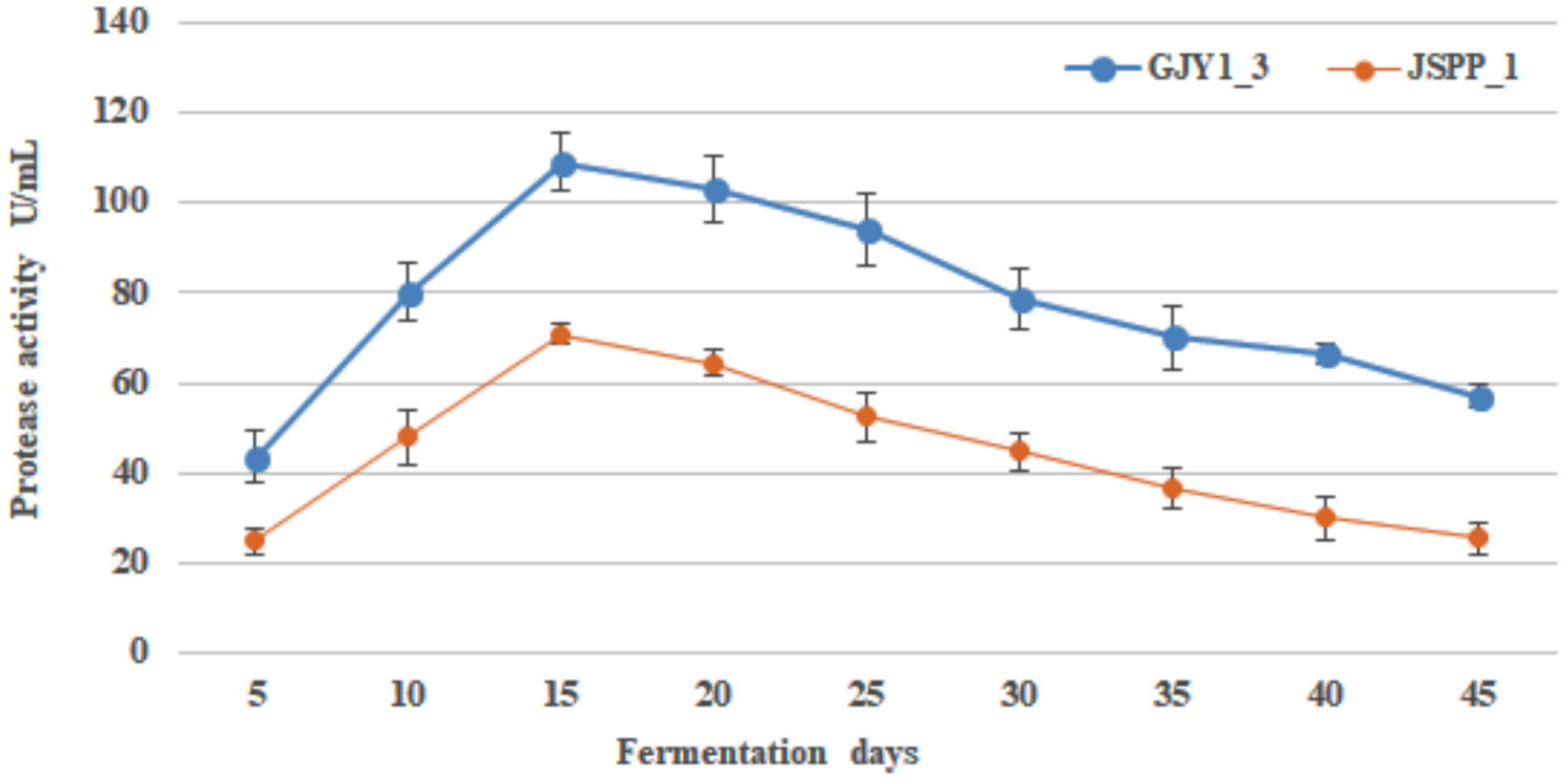
Changes in the protease activity of both strains over the fermentation time.

**Figure 2:**
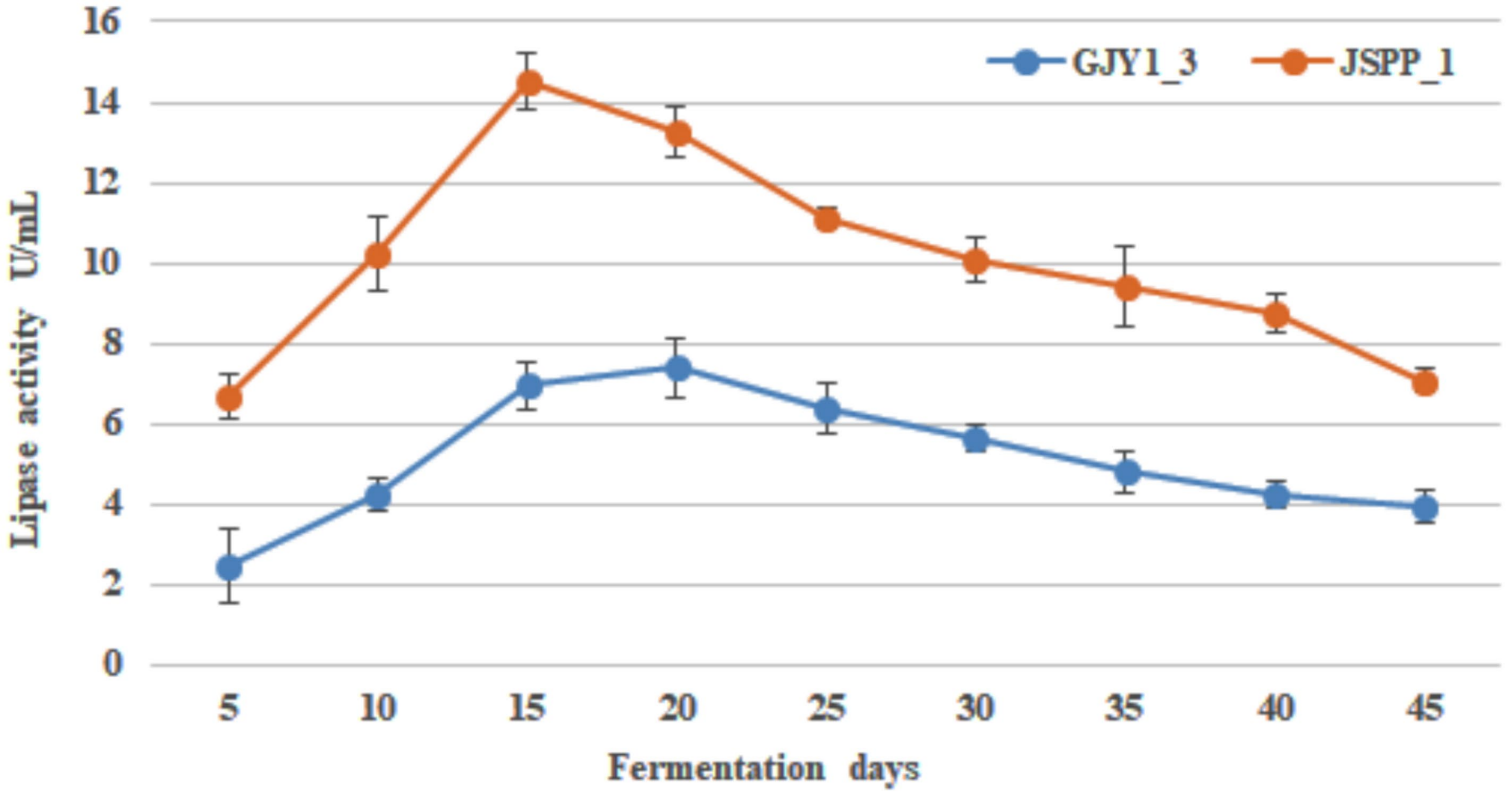
Changes in the lipase activity of both strains over the fermentation time.

The changes of protease and lipase activities of *P. chrysogenum* GJY1_3 and JSPP6_1 are shown in Figures 1, 2 and Tables 1, 2.

**Table 1:** Protease activity of the two strains.

Fermentation days	GJY1_3 U/mL	JSPP6_1 U/mL
5	43.48 ± 5.71	24.89 ± 3.07
10	80.17 ± 6.24	48.11 ± 6.17
15	108.95 ± 6.38	70.79 ± 2.13
20	102.79 ± 7.27	64.51 ± 3.11
25	94.16 ± 7.98	52.41 ± 5.61
30	78.49 ± 6.81	44.72 ± 4.16
35	70.11 ± 7.23	36.57 ± 4.32
40	66.34 ± 2.11	30.11 ± 4.83
45	57.18 ± 2.41	25.67 ± 3.65

**Table 2:** Lipase activity of the two strains.

Fermentation days	GJY1_3 U/mL	JSPP6_1 U/mL
5	2.47 ± 0.91	6.71 ± 0.56
10	4.26 ± 0.41	10.24 ± 0.94
15	6.97 ± 0.57	14.52 ± 0.68
20	7.41 ± 0.75	13.26 ± 0.61
25	6.41 ± 0.64	11.13 ± 0.23
30	5.65 ± 0.35	10.07 ± 0.56
35	4.84 ± 0.51	9.43 ± 0.97
40	4.26 ± 0.31	8.78 ± 0.48
45	3.97 ± 0.43	7.09 ± 0.31

As can be seen from the figure, the protease activity of GJY1_3 was significantly higher than that of JSPP6_1, and both strains reached the maximum value at 15 days of fermentation, which were 108.95 ± 6.38 and 70.79 ± 2.13 U/mL, respectively. The lipase activity of JSPP6_1 was higher than that of GJY1_3, and reached the maximum value at the 15th day of fermentation, which was 14.52 ± 0.68 U/mL, while that of GJY1_3 was 6.97 ± 0.57 U/mL. Subsequently, the growth rate of lipase activity slowed considerably and reached a maximum of 7.41 ± 0.75 U/mL at 20 days of fermentation.

Throughout the whole fermentation, the two strains grow rapidly and produce spores quickly. On the 5th day of fermentation, when the spores were initially produced, protease and lipase were detected a very high level. The protease and lipase activities of the two strains increased rapidly with the vigorous growth of the strains, the macromolecular nutrients in the soybean were fully decomposed, and the soybean paste became more viscous at this time, with a delicious smell. In the final stage, due to cell aging and metabolic stagnation, the activity of both enzymes gradually decreased. In view of the vigorous growth of the two strains, both showed good protease and lipase activities, indicating that both strains had excellent fermentation potential.

Many species of *Penicillium* are often used in the fermentation of certain foods, such as sausages, white mold cheese, blue mold cheese, etc. These *Penicillium* are used to improve the quality, texture, flavor and nutrition of food (Frisvad, 2014). *Penicillium chrysogenum* can produce penicillin and a variety of enzymes, which are often used in antibiotic production, but there are few reports on its application in fermented foods. *P. chrysogenum* has a high frequency of separation in the air and a rapid growing rate. Early in the fermentation of meat, it plays a role of rapid colonization and preventing other contaminated fungi from the air. During fermentation, *P. chrysogenum* can produce a variety of enzymes, such as protease, lipase, etc., which can decompose macromolecular of protein, fat, etc., reduce the hardness of meat, and improve the quality and taste of meat. At the same time, the growth of *P. chrysogenum* leads to the increase of non-protein nitrogen value and volatile substances in the catabolism of amino acids and fatty acids, and the increase of aroma intensity, giving special flavor to meat (Benito et al., 2005; Benito et al., 2003). Because of its unique fermentation potential and abundant secondary metabolites, *P. chrysogenum* has been used to ferment tea, duck meat and other foods. At present, there are fewer reports on the use of *P. chrysogenum* in fermenting soybean paste.

### 3.2 Different metabolites in fermentation of two strains

**Figure 3:**
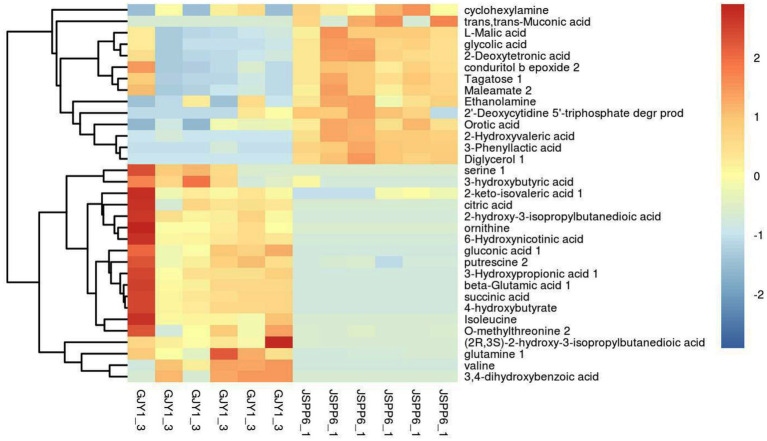
Heatmap of different metabolites in fermentation of GJY1_3 and JSPP6_1.

**Figure 4:**
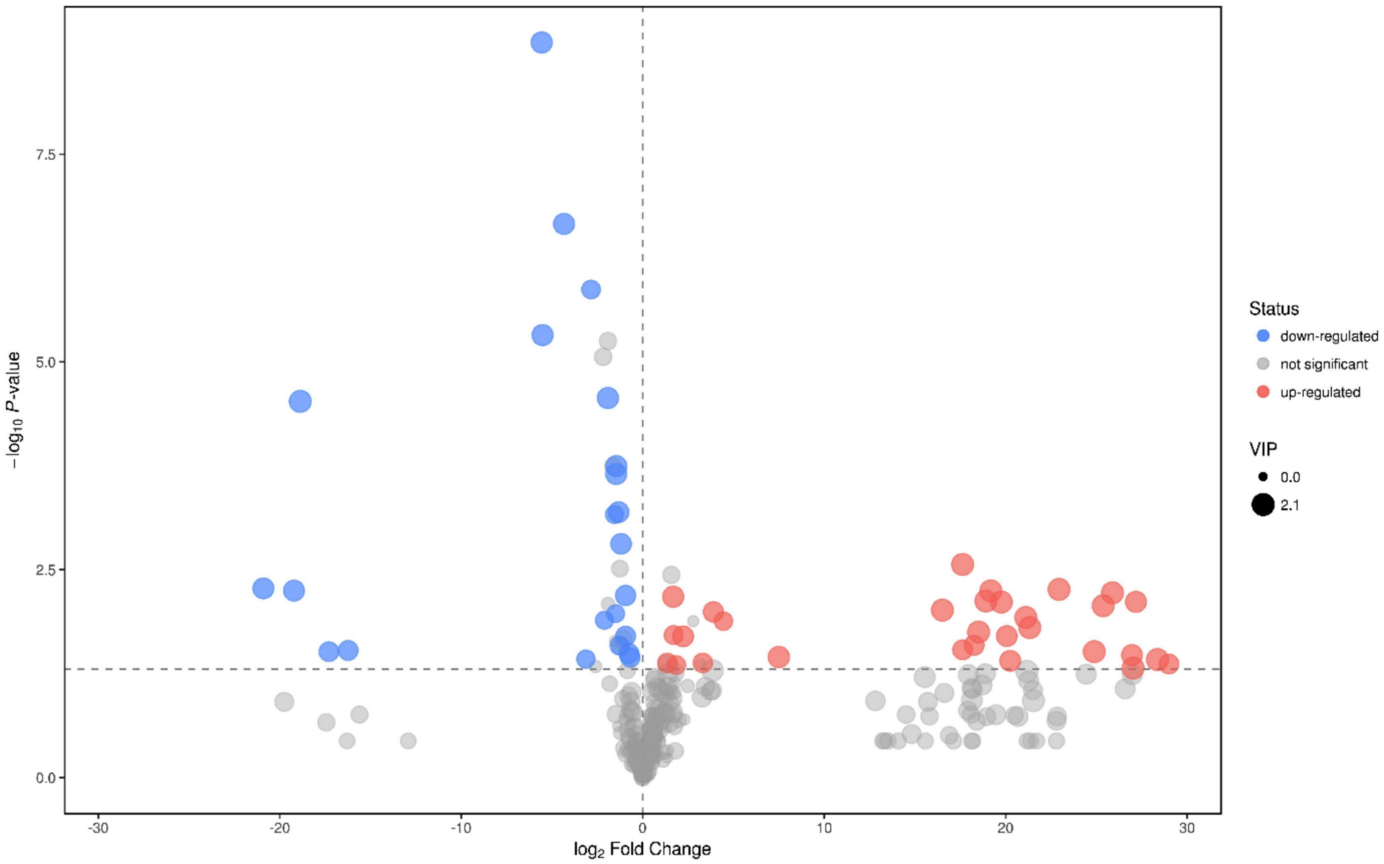
Volcano plot of GJY1_3 and JSPP6_1. (Each point represents a metabolite, and the point color represents the final screening result. The significantly upward-regulated metabolite is red, the significantly downward-regulated metabolite is blue, and the non-significant metabolite is grey).

When fermenting for 10–20 days, strains GJY1_3 and JSPP6_1 were the most vigorous in growth and metabolism, and the two enzyme activities had reached the maximum value in about 15 days. In order to know the difference of metabolites in fermentation of the two strains, the samples were collected for the study of metabolomics after 15 days of fermentation. Following the qualitative and relative quantitative analysis of fermentation products, the original data were processed by denoising, filtering and normalization. At the same time, the *p*-value of Student’s *t*-test was less than 0.05, and the variable importance in the projection (VIP) of the first principal component of OPLS-DA model was greater than 1 as the card value standard to screen differential metabolites. A total of 53 differential metabolites were achieved, of which 33 are known, and screening results are shown in Figures 3, 4.

#### 3.2.1 Amino acids

The relative contents of ornithine, serine, valine, isoleucine, glutamine, glutamate, and methylthreonine in GJY1 were significantly higher, which is directly associated with their higher protease activity. Proteases hydrolyze soy protein to release free amino acids such as glutamine and glutamate, reflecting active nitrogen metabolism. These amino acids serve as substrates for protein synthesis and an intermediate in the tricarboxylic acid cycle (TCA), thereby promoting energy metabolism and amino acid synthesis.

#### 3.2.2 Organic acids and anhydrides

There are 18 types of organic acids and anhydrides identified. In JSPP6_1, the relative content of 8 types of organic acids and anhydrides showed an upward trend, including malic acid, glycolic acid, hydroxyvaleric acid, deoxytetronic acid, phenyllactic acid, trans,trans-muconic acid, orotic acid, and maleamate. In GJY1, the relative contents of 10 organic acids increased, including succinic acid, citric acid, ketoisovaleric acid, hydroxypropionic acid, dihydroxybenzoic acid, 2-hydroxy-3-isopropylsuccinic acid, 3-hydroxybutyric acid, (2R, 3S)-2-hydroxy-3-isopropylsuccinic acid, gluconic acid, and 6-hydroxynicotinic acid. The differences in organic acid profiles between the two strains may result from preferences in carbon source utilization; for instance, GJY1 tends to utilize protein-based carbon sources, whereas JSPP6_1 prefers lipid-based carbon sources.

#### 3.2.3 Alcoholic substances

The enrichment of ethanolamine, cyclohexene tetraol, and diglycerol in JSPP6_1 correlates with its high lipase activity. Lipase catalyzes the hydrolysis of soybean fat, producing glycerol and fatty acids. Glycerol can be further converted into diglycerin or cyclic alcohols, while fatty acids undergo β-oxidation to produce aromatic alcohols, enhancing the flavor profile of soybean paste.

### 3.3 Metabolism pathways of differential metabolites

**Figure 5:**
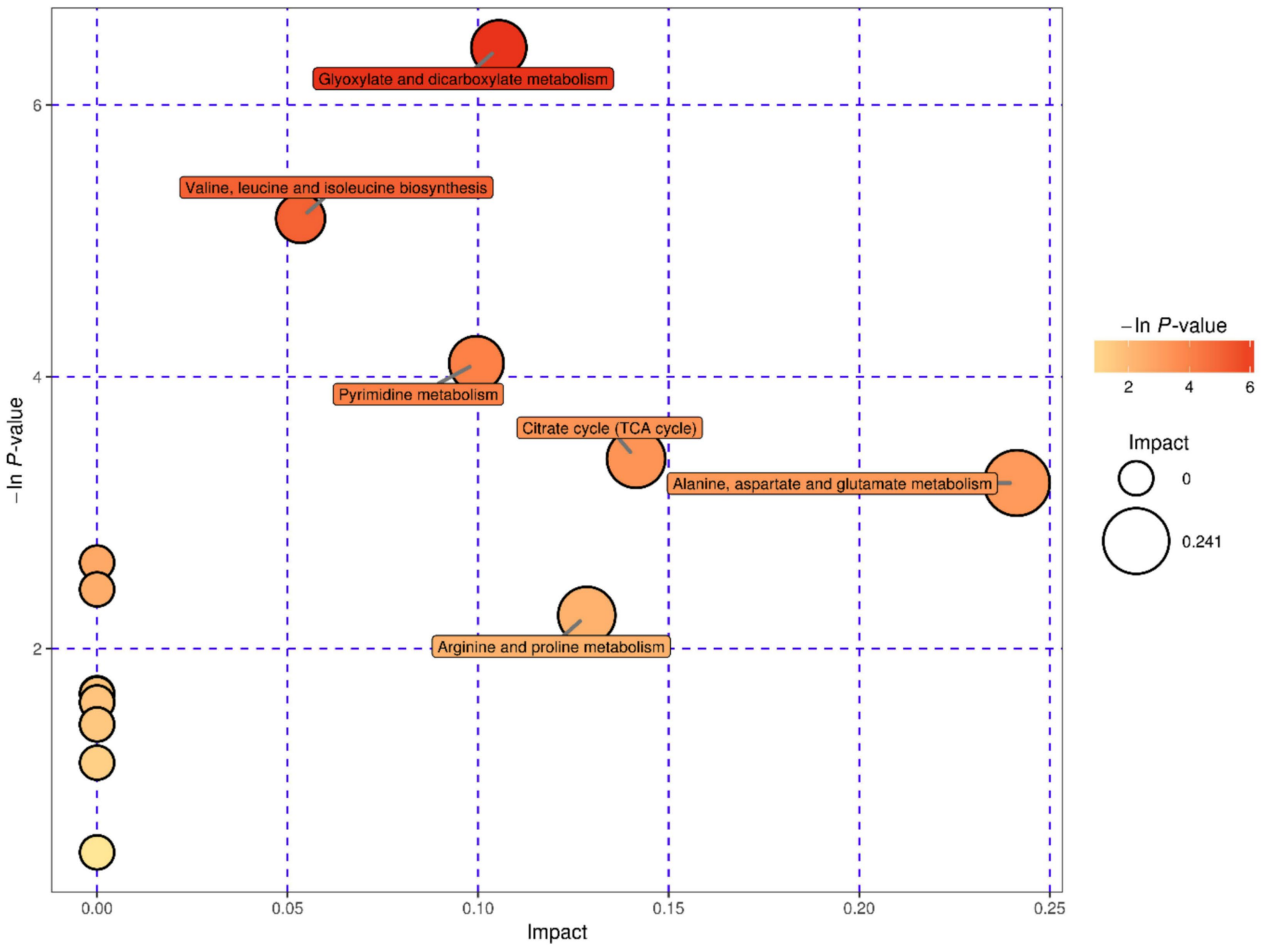
Analysis of principal metabolic pathways of GJY1_3 and JSPP6_1.

**Figure 6:**
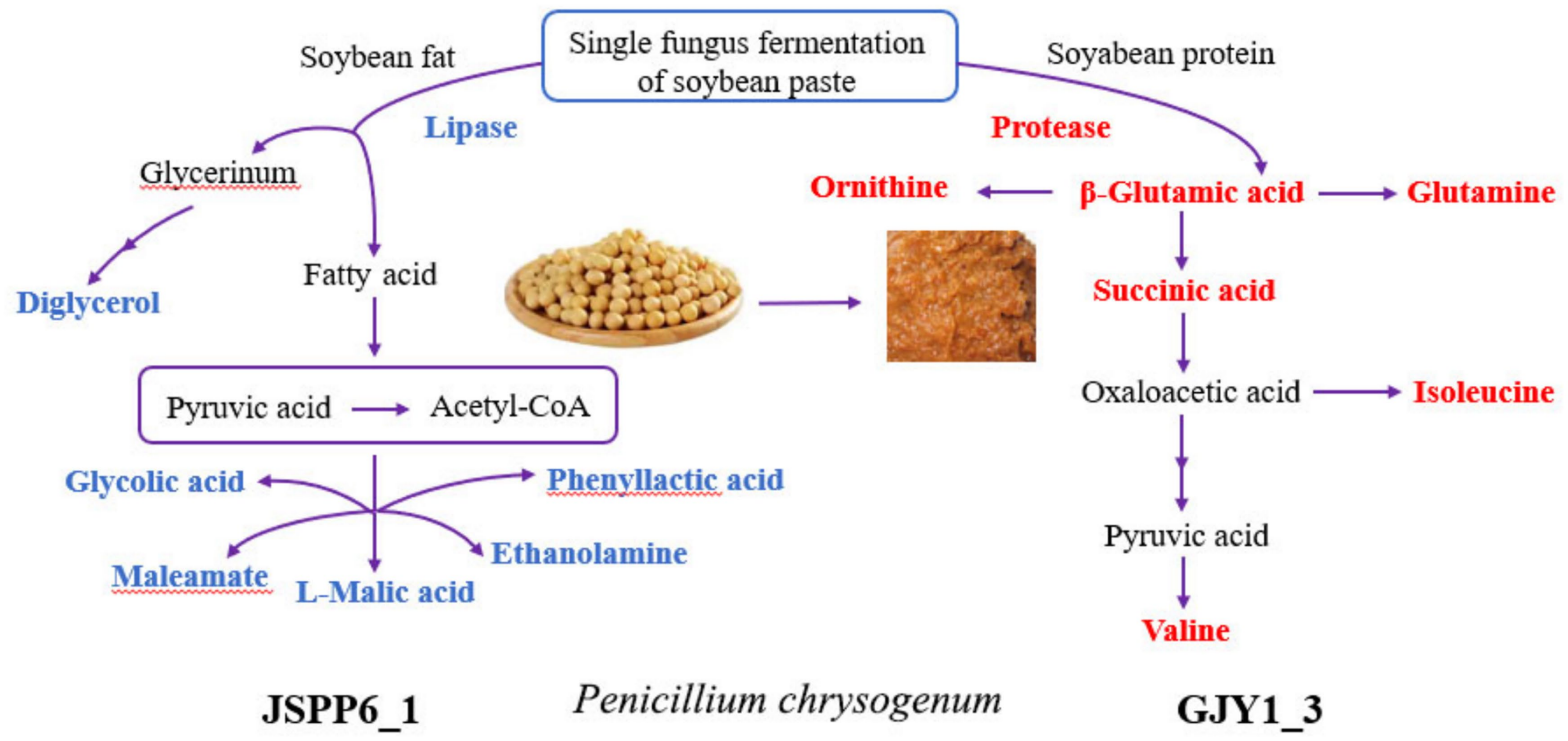
Diagram of enzymatic activities and partial differential metabolites in soybean paste fermentation of GJY1_3 and JSPP6_1. (The red substance indicates metabolites with higher relative levels of GJY1_3; the blue substance indicates metabolites with higher relative levels of JSPP6_1).

The 33 known differential metabolites were matched in the KEGG database to obtain the metabolic pathways involved in these metabolites. These metabolic pathways had been enriched and analyzed topologically to further filter key pathways with the highest correlation with metabolite difference (Xia et al., 2015; Kanehisa and Goto, 2000; Kanehisa et al., 2016). A total of 17 metabolic pathways were obtained in the study, of which six were highlighted important metabolic pathways, including glyoxylate and dicarboxylate metabolism, valine, leucine and isoleucine biosynthesis, pyrimidine metabolism, citrate cycle, arginine and proline metabolism and alanine, aspartate and glutamate metabolism. See Table 3 and Figures 5, 6 for more information on important metabolism pathways.

**Table 3:** Analysis of the principal metabolic pathways of GJY1_3 and JSPP6_1.

Pathway	Total	Hits	Raw *p*	−ln(*p*)	Holm adjust	FDR	Impact
Glyoxylate and dicarboxylate metabolism	17	3	0.0016	6.4214	0.1415	0.1415	0.1054
Valine, leucine and isoleucine biosynthesis	26	3	0.0057	5.1647	0.4915	0.2486	0.0534
Pyrimidine metabolism	38	3	0.0166	4.1001	1	0.4806	0.0996
Citrate cycle (TCA cycle)	20	2	0.0335	3.3977	1	0.6954	0.1415
Alanine, aspartate and glutamate metabolism	22	2	0.0399	3.2198	1	0.6954	0.2414
Arginine and proline metabolism	38	2	0.1058	2.2462	1	1	0.1285

Pathway is the name of metabolic pathway. Total is the number of metabolites in the pathway. Hits is the number of differential metabolites hitting this pathway. Raw *p* is the *p*-value of the enrichment analysis of the metabolic pathway. −ln (*p*) is the negative logarithm of the *p*-value with e as the base (negative natural logarithm). Holm adjust is the *p*-value corrected by multiple hypothesis test using Holm–Bonferroni method. FDR is the *p*-value corrected by multiple hypothesis test using the false discovery rate (FDR) method. Impact is the impact value of the metabolic pathway topology analysis. It is usually thought that the larger the impact and −ln *p*-value values, the better the correlation.

#### 3.3.1 Differences in carbon source utilization and core metabolic pathways

GJY1 produces proteases that efficiently decompose soy protein, thereby activating the metabolic pathways of alanine, aspartate, and glutamate. This significantly upregulates the levels of amino acids such as glutamate and glutamine and generates organic acids like succinic acid and citric acid via the TCA cycle, providing essential energy and nitrogen sources for its growth. In contrast, JSPP6_1 utilizes lipase to degrade soybean fat, with its metabolic network primarily focused on glyoxylate and dicarboxylate metabolism. This leads to the accumulation of organic acids such as malic acid and maleic anhydride, as well as alcohol compounds like glycerol and aminoethanol.

#### 3.3.2 Synthesis of branched chain amino acids

The enrichment of valine, leucine, and isoleucine biosynthesis pathways in GJY1 correlates with its high content of valine and isoleucine. These branched-chain amino acids can be degraded into ketone acids (e.g., pyruvic acid → acetyl-CoA), which further participate in the TCA cycle or contribute to flavor substance synthesis. Meanwhile, the active pyrimidine metabolism in JSPP6_1 likely supports nucleotide synthesis. Additionally, the fatty acids (e.g., phenyllactic acid and hydroxyvaleric acid) and aromatic alcohols (e.g., cyclohexene tetraol) produced by its lipolysis directly enhance the lipid aroma and complex flavor profile of soybean paste.

The amino acid metabolism in GJY1 dominates the accumulation of umami substances (e.g., glutamic acid), while the lipid metabolism in JSPP6_1 generates flavor-enhancing substances such as malic acid (for acidity regulation) and aromatic alcohols (for flavor enhancement). The complementary metabolisms of these two strains synergistically balance umami, acidity, and aroma, thereby improving the overall quality of soybean paste.

## 4 Conclusion

Two strains of *Penicillium chrysogenum*, GJY1_3 and JSPP6_1, exhibited significant differences in product characteristics during soybean paste fermentation. Strain GJY1_3, characterized by high protease activity, significantly increased the umami amino acid content in soybean paste via the TCA cycle and associated amino acid metabolic pathways (e.g., glutamate and glutamine synthesis), thereby enriching the flavor profile of the product. In contrast, strain JSPP6_1, with its remarkable lipase activity, effectively degraded lipids to produce fatty acids, contributing to the distinctive flavor of soybean paste. Metabolomics analysis further elucidated variations in multiple metabolic pathways between the two strains, resulting in pronounced differences in the abundance of amino acids and organic acid metabolites. Leveraging the complementary functionalities of these two strains, mixed fermentation can synergistically enhance the nutritional diversity and flavor complexity of soybean paste, offering novel insights for the development of functional fermented food. Studies have demonstrated that *Penicillium chrysogenum* produces mycotoxins, including PR-toxin, roquefortine C, and secalonic acids, which exhibit cytotoxic effects, disrupt enzymatic biosynthesis, and display teratogenic potential (Visagie et al., 2014). Despite the potential risk of mycotoxin production by *P. chrysogenum*, the screened strains GJY1_3 and JSPP6_1 exhibited remarkable enzymatic activity and metabolic potential during soybean paste fermentation. With the increasing application of *P. chrysogenum* in the food industry, it is crucial to develop a dynamic toxin monitoring system and adopt biotechnological breeding strategies to ensure its safe and effective use in food fermentation processes.

## Data Availability

The original contributions presented in the study are included in the article/supplementary material, further inquiries can be directed to the corresponding author.

## Footnotes

^1^ http://www.genome.jp/kegg/

^2^ http://www.nist.gov/index.html
